# Vasoinhibins Prevent Bradykinin-Stimulated Endothelial Cell Proliferation by Inactivating eNOS *via* Reduction of both Intracellular Ca^2+^ Levels and eNOS Phosphorylation at Ser^1179^

**DOI:** 10.3390/ph4071052

**Published:** 2011-07-20

**Authors:** Stéphanie Thebault, Carmen González, Celina García, David Arredondo Zamarripa, Gabriel Nava, Luis Vaca, Fernando López-Casillas, Gonzalo Martínez de la Escalera, Carmen Clapp

**Affiliations:** 1 Instituto de Neurobiología, Universidad Nacional Autónoma de México (UNAM), Campus UNAM-Juriquilla, Querétaro 76230, Mexico; E-Mails: cgonzalez.uaslp@gmail.com (C.G.); anilec94@hotmail.com (C.G.); david_arrz@hotmail.com (D.A.Z.); ganava@myway.com (G.N.); gmel@servidor.unam.mx (G.M.E.); clapp@servidor.unam.mx (C.C.); 2 Instituto de Fisiología Celular, Universidad Nacional Autónoma de México (UNAM), Ciudad Universitaria, Del. Coyoacán, México, D.F., 04510, Mexico; E-Mails: lvaca@ifc.unam.mx (L.V.); fcasilla@ifc.unam.mx (F.L.-C.)

**Keywords:** vasoinhibins, 16kDa-prolactin, bradykinin, endothelial nitric oxide synthase, calcium mobilization, transient receptor potential channels

## Abstract

Vasoinhibins, a family of antiangiogenic peptides derived from prolactin proteolysis, inhibit the vascular effects of several proangiogenic factors, including bradykinin (BK). Here, we report that vasoinhibins block the BK-induced proliferation of bovine umbilical vein endothelial cells. This effect is mediated by the inactivation of endothelial nitric oxide synthase (eNOS), as the NO donor DETA-NONOate reverted vasoinhibin action. It is an experimentally proven fact that the elevation of intracellular Ca^2+^ levels ([Ca^2+^]_i_) upon BK stimulation activates eNOS, and vasoinhibins blocked the BK-mediated activation of phospholipase C and the formation of inositol 1,4,5-triphosphate leading to a reduced release of Ca^2+^ from intracellular stores. The [Ca^2+^]_i_ rise evoked by BK also involves the influx of extracellular Ca^2+^ *via* canonical transient receptor potential (TRPC) channels. Vasoinhibins likely interfere with TRPC-mediated Ca^2+^ entry since La^3+^, which is an enhancer of TRPC4 and TRPC5 channel activity, prevented vasoinhibins from blocking the stimulation by BK of endothelial cell NO production and proliferation, and vasoinhibins reduced the BK-induced increase of TRPC5 mRNA expression. Finally, vasoinhibins prevented the BK-induced phosphorylation of eNOS at Ser^1179^, a post-translational modification that facilitates Ca^2+^-calmodulin activation of eNOS. Together, our data show that vasoinhibins, by lowering NO production through the inhibition of both [Ca^2+^]_i_ mobilization and eNOS phosphorylation, prevent the BK-induced stimulation of endothelial cell proliferation. Thus, vasoinhibins help to regulate BK effects on angiogenesis and vascular homeostasis.

## 1. Introduction

Vasoinhibins, originating from the proteolysis of the peptide hormone prolactin, have emerged as natural inhibitors of retinal angiogenesis [1]. Vasoinhibins help maintain retinal avascularity [2], and diabetic retinopathy is associated with reduced levels of vasoinhibins in the retina [3] and in the circulation [4]. Furthermore, the intravitreal injection of vasoinhibins blocks the aberrant retinal vasodilation and vasopermeability induced by diabetes and by the intravitreal injection of vascular endothelial growth factor (VEGF) [5]. Vasoinhibins block several other vascular effects of VEGF (for review, see [6]) and reduce the diabetes-induced increase in retinal vasopermeability in a manner equivalent to that of VEGF immunodepletion [5]. Anti-VEGF agents have been approved to treat age-related macular degeneration, and are in clinical trials for diabetic macular edema and diabetic retinopathy [7]. It is important to note that proangiogenic factors other than VEGF are under intensive investigation in relation to diabetic retinopathy. In particular, inhibition of the bradykinin (BK) signaling pathway is a therapeutic option [8-10], because BK levels are elevated in the vitreous of patients with diabetic retinopathy [11], and BK increases the permeability, dilation, proliferation, and survival of blood vessels [12,13]. Vasoinhibins prevent BK-induced vasodilation [14], but whether they inhibit the vasoproliferative effect of BK is unknown.

It is well established that nitric oxide (NO) produced by the activation of endothelial NO synthase (eNOS) mediates most vascular actions of BK [15], and vasoinhibins block BK-induced eNOS activation [14]. In its basal state, eNOS has a low affinity for Ca^2+^/calmodulin, and its activation depends on the elevation of intracellular Ca^2+^ levels. The stimulation of B2 receptors by BK initiates the classical G(q) protein-coupled receptor activation of phospholipase C-β (PLC-β) that catalyzes the formation of inositol 1,4,5-trisphosphate (IP_3_) and diacylglycerol, leading to a biphasic increase in intracellular free Ca^2+^ concentration ([Ca^2+^]i). The initial transient component reflects an IP_3_-induced release of Ca^2+^ from endoplasmic reticulum, whereas the longer-lasting elevation reflects Ca^2+^ influx from the extracellular space [16,17]. Ca^2+^ influx in response to activation of PLC-β is thought to involve canonical transient receptor potential (TRPC) channels [18]. The mammalian TRPC family consists of seven members, designated TRPC1–TRPC7 [19,20], and TRPC6 contributes to the BK-induced increase in intracellular Ca^2+^ in some endothelial cells [21]. Another mechanism by which BK activates eNOS is *via* protein kinase A-dependent phosphorylation at serine^1179^ [22], a posttranslational modification that facilitates the Ca^2+^/calmodulin binding to eNOS [23,24].

Vasoinhibins interfere with the mobilization of intracellular Ca^2+^ evoked by BK [14] and block VEGF-induced eNOS phosphorylation at serine^1179^ [5]. In this study, we investigated whether vasoinhibins inhibit BK-induced endothelial cell proliferation by interfering with BK-induced activation of eNOS *via* PLC-β activation, IP_3_ formation, Ca^2+^ release from intracellular stores, TRPC-mediated Ca^2+^ entry, and eNOS phosphorylation at serine^1179^. The results presented here provide a complex and concertized series of events leading to the modulation of endothelial cell proliferation by vasoinhibins.

## 2. Experimental

### 2.1. Reagents

Bradykinin, thapsigargin, SKF-96365, ruthenium red, Gd^3+^, La^3+^, and okadaic acid were purchased from Sigma (St. Louis, MO, USA). Bovine PRL (BIO grade) was obtained from the National Hormone and Pituitary Program (NHPP, Torrance, CA, USA). (*Z*)-1-[*N*-(2-Aminoethyl)-*N*-(2-ammonioethyl)amino]diazen-1-ium-1,2-diolate or DETA-NONOate was purchased from Alexis Corporation (San Diego, CA, USA). Recombinant human vasoinhibins (corresponding to both 16 and 18 kDa fragments of prolactin) were produced by site-directed mutagenesis using a baculovirus expression system as described [25]. Negative controls included aliquots of recombinant vasoinhibins that were inactivated by incubation for 30 min at 90 °C, followed by centrifugation for 60 min at 2500 × *g* to remove denatured proteins.

### 2.2. Bovine Umbilical Vein Endothelial Cell (BUVEC) Culture

BUVEC obtained and cultured as described [26] were routinely grown in F12K medium (A1963, AppliChem GMBH, Darmstadt, Germany) supplemented with 10% FBS and 50 U/mL penicillin/streptomycin. For all experiments, cells were incubated in 0.5% FBS-culture medium for 24 h prior treatments.

### 2.3. Cell Proliferation Assay

Cell proliferation was first assessed by [^3^H]thymidine incorporation (Amersham International plc, Cardiff, UK), and once validated, we used the reduction of 3-(4,5-dimethylthiazol-2-yl)-2,5-diphenyltetrazolium bromide (MTT) as an index of proliferation to avoid radioisotopes. The ^3^H-thymidine incorporation assay consisted in seeding cells in 48-well plates (Nunc) at an initial density of 5000 cells/well and subjecting them to a series of treatments (all in 200 μL of complete culture medium containing 10% FBS) that included a 48 h-incubation with BK (10 μM) combined or not with increasing doses of vasoinhibins (10, 20, 40, and 60 nM), and in the presence or the absence of DETA-NONOate (10 μM), as indicated. When required, treatments were performed in Ca^2+^-free medium obtained by adding 10 mM EDTA to the standard F12K medium, which normally contains 0.4 mM Ca^2+^. Twenty-four h before ending the treatment, 1 μL of ^3^H-thymidine per well was added. After the 48-h treatment, wells were washed thrice in 5% trichloroacetic acid, with the last wash for 20 min at 4 °C. Next, 250 μL boiling 0.25 N NaOH was added, and samples were transferred to scintillation vials for quantification. For the MTT assay, cells were seeded in 96-well plates (Nunc) at an initial density of 2500 cells/well and treated for 48 h with 10% FBS-culture medium supplemented or not with BK (10 μM), vasoinhibins (60 nM), and the nonselective TRP channel blockers SKF-96365 (10 μM) [27,28], ruthenium red (10 mM), or Gd^3+^ (1 μM), as indicated. La^3+^ (10 μM), known to enhance the activity of TRPC4 and 5 [20], was also tested. In another group of experiments, BUVEC were incubated with vasoinhibins (60 nM) and BK (10 μM) in the presence or in the absence of okadaic acid (50 nM) for 48 h. Next, cells were incubated with MTT (500 mg/mL, Sigma-Aldrich) at 37 °C for 4 h, and the formazan precipitate was solubilized with 0.4 N HCl-10 % SDS for 30 min at room temperature and quantified by measuring absorbance at 570 nm.

### 2.4. PLC Activity Measurement

PLC activity was measured according to the method of Hofmann and Majerus [29]. Reaction mixtures contained 280 μM phosphatidylinositol, 30,000 dpm of PIP_2_-(myo-inositol-2-^3^H(N)), 1 mg/mL sodium deoxycholate, 1 mM CaCl_2_, 50 mM HEPES (pH 7.0), and BUVEC extracts. After incubation for 15 min at 37 °C, BUVEC extracts were incubated for 5 min with vasoinhibins (60 nM) prior to a 2-min incubation with BK (10 μM). A two-min incubation with BK, vasoinhibins, or basal medium (ctl) was also tested. The reaction was stopped with 1 mL of chloroform/methanol (1:2 v/v), followed by 0.3 mL of 1 N HCl containing 5 mM EGTA. After centrifugation for 10 min at 3000 × *g*, the supernatant was removed for liquid scintillation counting. Protein content was measured using bicinchoninic acid (TM) (American Radiolabeled Chemicals, Inc., St. Louis, MO, USA).

### 2.5. D-myo-IP_3_ Measurement

The accumulation of IP_3_ was measured by the receptor displacement assay described by Challiss *et al.* [30]. Briefly, BUVEC were incubated for 2 min with basal medium (ctl), vasoinhibins (60 nM) or BK (10 μM), this latter being preceded or not by a 5-min incubation with vasoinhibins. The reaction was stopped after periods of 1 and 2 min by the addition of ice-cold perchloric acid to extract IP_3_. Following a 20-min incubation on ice, the tubes were centrifuged at 2000 × *g* for 15 min; the supernatants were neutralized with 5 N KOH. IP_3_ content was assayed using the inositol-1,4,5-triphosphate [^3^H] radioreceptor assay procedure (Perkin Elmer Life Sciences, Inc., Boston, MA, USA), a competitive ligand binding assay.

### 2.6. Intracellular Ca^2+^ Measurement

Intracellular Ca^2+^ was measured using an Aminco-Bowman Series-2 luminescence spectrometer with a 150-W xenon source (Rochester, NY, USA). BUVEC were mechanically dispersed after a 2-min incubation with 0.01% trypsin-EDTA solution, centrifuged, and resuspended at a final density of 10^6^ cells/mL in HBSS (Hanks balanced salt solution) containing (in mM): 140 NaCl, 5 KCl, 1 MgCl_2_, 2 CaCl_2_, 0.3 Na_2_HPO_4_, 0.4 KH_2_PO_4_, 4 NaHCO_3_, 5 glucose, and 10 HEPES (adjusted to pH 7.4 with NaOH). Cells were loaded with Fura-2 by exposure to 2 μM Fura-2 AM (Molecular probes, Eugene, OR, USA) at 37 °C for 1 h in HBSS. After incubation, cells were washed three times in HBSS. Cell suspensions were then incubated under constant perfusion with Ca^2+^-free or 2 mM Ca^2+^-containing HBSS containing BK (10 μM) or thapsigargin (Tg, 1 μM), as indicated. Ca^2+^-free medium was prepared by omitting CaCl_2_ and adding 10 mM EGTA. Prior to BK perfusion, BUVEC were incubated for 2 min with vasoinhibins (60 nM), heat-inactivated vasoinhibins, or prolactin (10 nM). Similarly, prior to Tg perfusion, BUVEC were incubated for 2 min with vasoinhibins (60 nM). Fura-2 fluorescence, at an emission wavelength of 510 nm, was recorded by exciting the probe alternately at 340 and 380 nm. Cytosolic [Ca^2+^] was derived from the 340 nm/380 nm signal ratio using the Grynkiewicz equation [31]. All reagents were diluted to their final concentration in HBSS and applied with a perfusion system. Data were accumulated from at least three measurements for each experiment.

### 2.7. Evaluation of NO Production

NO production was assessed by measuring the accumulation of nitrites, a stable end product of NO metabolism, in the supernatant of BUVEC. Cells were grown in 12-well plates until 80% confluence and then incubated for 1 h with BK (10 μM) combined or not with vasoinhibins (60 nM), and in the presence or the absence of La^3+^ (10 μM). The culture media were collected. The amount of nitrites was determined spectrophotometrically by the Griess reagent. A 100-μL aliquot of sample was added to 100 μL of Griess reagent (1% sulfanilamide, 0.1% naphthyl ethylenediamine dihydrochloride, and 2% phosphoric acid). After a 10-min incubation at room temperature, the absorbance was measured at 550 nm with a Microplate Reader (Bio-Tek Instruments, Winooski, VT, USA), and the NO concentration in samples was determined using a curve calibrated with NaNO_2_ standards.

### 2.8. Evaluation of eNOS Activity by L-Citrulline Assay

eNOS activity was determined by L-citrulline assay in BUVEC grown in 12-well plates until 80% confluence that were then pretreated with okadaic acid (50 nM) for 10 min, then vasoinhibins (60 nM) for 1 h and followed or not by BK (10 μM) for 10 min. Protein concentration was evaluated using the Bradford assay (Bio-Rad). The reaction was carried out in reaction buffer (50 mM HEPES, 1 mM NADPH, 1.25 mM CaCl_2_, 1 μM FAD, 1 μM FMN, and 10 μg/mL calmodulin) and initiated by adding 1 μCi/mL [^3^H]l-arginine and 50 μg of BUVEC protein in a final volume of 100 μL. After 1 h, ice-cold stop medium (50 mM HEPES, pH 5.5, and 4 mM EDTA) was added for 10 min. BUVEC extracts were then applied onto 1-mL columns of Dowex AG50WX8. [^3^H]L-citrulline was eluted with 1 mL water and quantified by liquid scintillation counting.

### 2.9. RT-PCR

Total RNA from BUVEC was isolated using TRIzol (GIBCO/BRL, Life Technologies, Breda, The Netherlands) prior to reverse transcription using Moloney murine leukemia virus reverse transcriptase (Promega, Madison, WI). cDNA was amplified using TRPC5 (Forward primer 5′-TGCAACTGTGTGGAGTGTGT-3′, reverse primer 5′-TGTGGTCATCTCGATGGTTGA-3′), TRPC6 (Forward primer 5′-GTCATGAATGCAGCTGACAGA-3′, reverse primer 5′-CTTTACATTCAGCCCATATCAT-3′), TRPM6 (Forward primer 5′-CCAAGCACCTTTTCCAAATTCT-3′, reverse primer 5′-TCCCAAGCCATTGCCAGATT-3′), TRPM7 (Forward primer 5′-GCCCCGTGAGGAGAATGTC-3′, reverse primer 5′-GTATTTCATGGCAAGACTTGCA-3′), actin (Forward primer 5′-CCATCATGAAGTGTGACGTTG-3′, reverse primer 5′-ACAGAGTACTTGCGCTCAGGA-3′), and L19 (Forward primer 5′-CGAAATCGCCAATGCCAACTC-3′, reverse primer 5′-TGCTCCATG AGAATCCGCTTG-3′), and subsequently analyzed by agarose gel electrophoresis. Because identical amounts of cDNA were added to the PCR reaction, we estimated the changes in transcript expression levels relative to those of housekeeping genes.

### 2.10. Immunoblotting

BUVEC were pretreated with vasoinhibins (60 nM) for 1 h followed by incubation with BK (10 μM) for 1, 5, or 10 min. Protein samples were denatured by incubation in Laemmli buffer, and then subjected to SDS-PAGE. Immunoblots were incubated with polyclonal anti-phospho-Ser^1179^-eNOS antibody (1:1000 dilution; catalog 9571; Cell Signaling Technology, Danvers, MA, USA) overnight, and the antigen-antibody complex was detected using HRP-coupled secondary antibodies (catalog 111-035-003; Jackson ImmunoResearch Laboratories Inc., West Grove, PA, USA) and enhanced chemiluminescence (ECL; Super-Signal West Pico Chemiluminescent Substrate; Pierce Biotechnology, Rockford, IL, USA). Membranes were then stripped and probed with monoclonal anti-eNOS (1:1,000 dilution; catalog 33–4500, Zymed Laboratories Inc., Invitrogen, Carlsbad, CA, USA) and the alkaline phosphatase secondary antibody kit (Bio-Rad, Hercules, CA, USA).

### 2.11. Statistical Analysis

Values are expressed as mean ± SEM. Statistical significance between groups was determined by analysis of variance (ANOVA) followed by unpaired Student's T-test. Differences in means with P < 0.05 were considered statistically significant. The statistical analysis was performed using the Sigma Stat 7.0 software (Systat Software Inc., San Jose, CA, USA).

## 3. Results and Discussion

### 3.1. Vasoinhibins Prevent NO-Dependent, BK-Stimulated BUVEC Proliferation by Impairing Ca^2+^ Homeostasis

**Figure 1:**
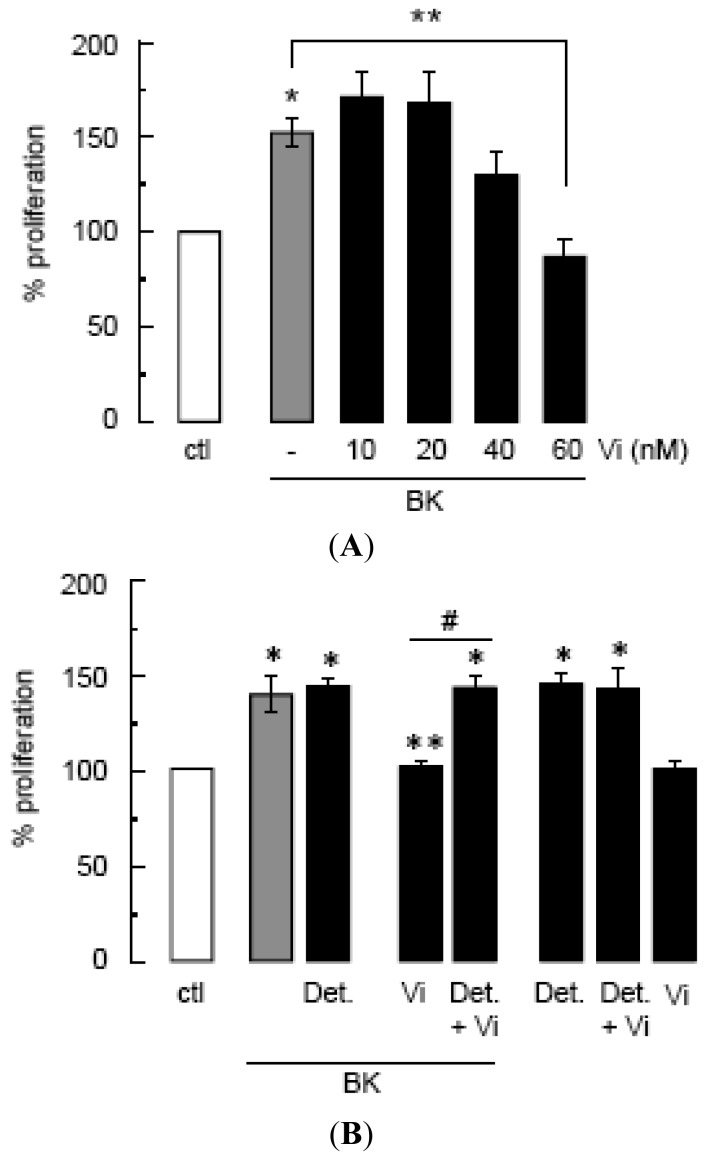
Vasoinhibins block the BK-induced proliferation of BUVEC in a NO-dependent manner.

Compared to untreated control cultures, BK induced a 50% increase in BUVEC proliferation that was significantly diminished by vasoinhibins at a dose of 60 nM (Figure 1A). The NO donor DETA-NONOate stimulated BUVEC proliferation to similar levels as BK, but it did not enhance BK action (Figure 1B) confirming first, that NO is a major mediator of BUVEC proliferation and second, that BK acts by promoting NO production. In contrast, DETA-NONOate prevented the vasoinhibin blockage of BK-stimulated BUVEC proliferation (Figure 1B). Vasoinhibins alone had no effect, and they could not prevent the stimulatory action of DETA-NONOate (Figure 1B), indicating that vasoinhibins signal upstream of NO production. Previous studies have unveiled eNOS activation as a target of vasoinhibin actions [5,14].

**Figure 2:**
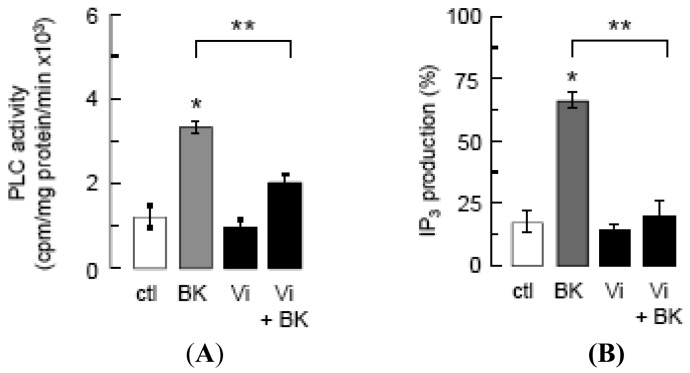
Vasoinhibins prevent the BK-mediated activation of PLC and the formation of IP_3_.

Inhibition of intracellular Ca^2+^ mobilization was postulated to mediate vasoinhibin blockage of eNOS activation [14], and BK mobilizes Ca^2+^ from intracellular stores by generating IP_3_ [15]. We show that vasoinhibins reduced BK-stimulated PLC activity (Figure 2A) and IP_3_ production (Figure 2B) to basal levels, but vasoinhibins alone had no effect on either parameter. Whether vasoinhibins interfere with BK stimulation of Gαq, Gαi, or B2 receptor proteins has not been determined yet. In this context, it is worth mentioning that vasoinhibins specifically bind to high-affinity saturable sites in endothelial cells [32] but their molecular nature remains to be unveiled so as to provide a proximal mechanism for vasoinhibin action.

**Figure 3:**
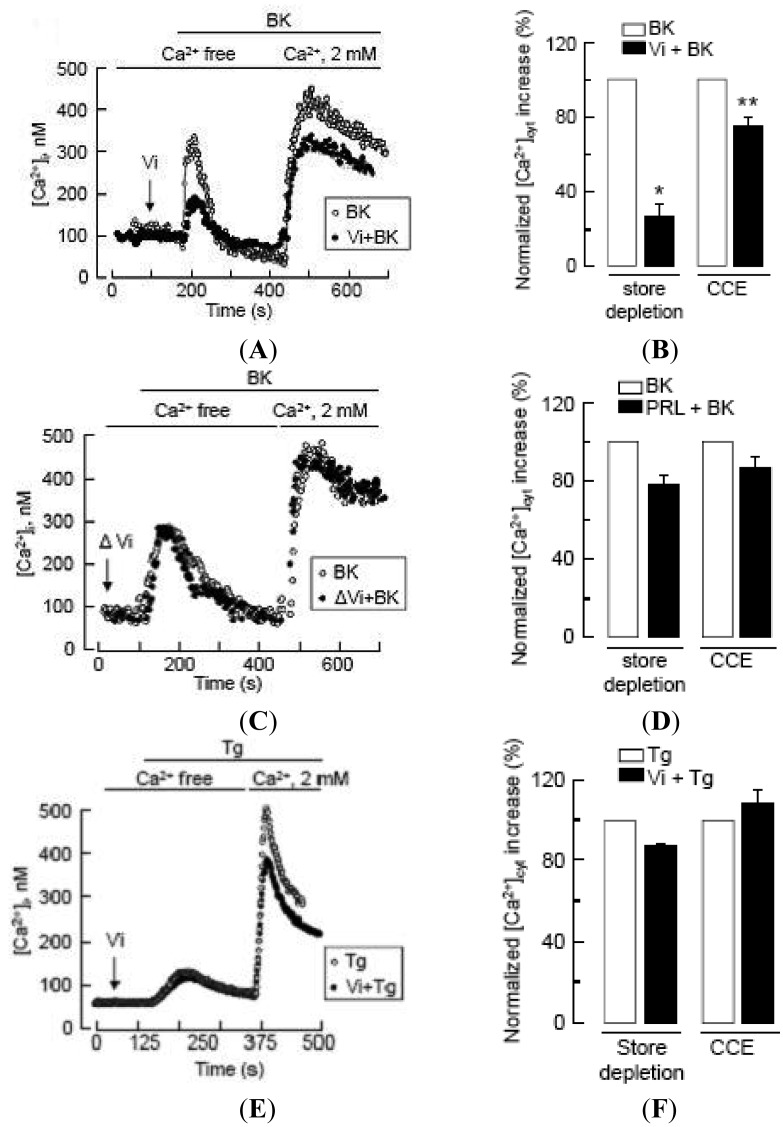
Vasoinhibins reduce both Ca^2+^ store depletion and capacitative Ca^2+^ entry (CCE) stimulated by BK in BUVEC.

IP_3_ can promote extracellular Ca^2+^ entry by depleting Ca^2+^ stores, a process known as capacitative Ca^2+^ entry [33,34], but also by an independent pathway [34,35]. In particular, BK can stimulate both capacitative [34] and non-capacitative Ca^2+^ entry in endothelial cells [21,36-38]. To define the action of vasoinhibins in BK-induced extracellular Ca^2+^ entry, we quantified the amount of Ca^2+^ released from the endoplasmic reticulum and measured the magnitude of the Ca^2+^ entry in response to the addition of 2 mM Ca^2+^ to the bath solution. Vasoinhibins clearly reduced the depletion of intracellular Ca^2+^ stores in response to BK, as previously shown [14], and they also reduced the BK-induced capacitative Ca^2+^ entry (Figures 3A,B). This effect was specific since heat-denatured vasoinhibins (Figure 3C) and full-length prolactin (Figure 3D) lacked action. Importantly, BUVEC cell perfusion with thapsigargin, a well-known inhibitor of sarcoplasmic and endoplasmic reticulum Ca^2+^ ATPase that depletes intracellular Ca^2+^ stores without affecting IP_3_ production, activated a Ca^2+^ influx through store-operated channels that was insensitive to vasoinhibins (Figures 3E,F). These data suggest that vasoinhibins can regulate the activity of plasma membrane channels independently of Ca^2+^ store depletion. These latter may be the BK-activated cation channels reported to mediate non-capacitative Ca^2+^ entry in endothelial cells [38].

**Figure 4:**
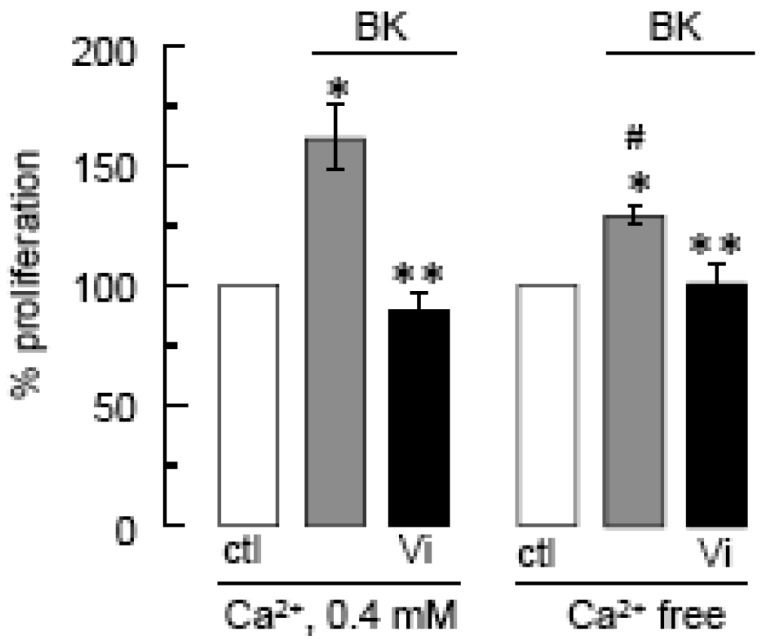
Impact of extracellular Ca^2+^ on BK-induced BUVEC proliferation and on the inhibitory effect of vasoinhibins.

The contribution of Ca^2+^ influx to vasoinhibin blockage of BK-induced BUVEC proliferation was examined next. Using the basal culture medium that contains 0.4 mM Ca^2+^, we can measure cell events that involve both Ca^2+^ entry and intracellular Ca^2+^ mobilization. The use of Ca^2+^-depleted medium eliminates Ca^2+^ influx, thereby helping to identify its contribution when compared to basal culture medium conditions. Figure 4 shows that BK required extracellular Ca^2+^ to exert its maximal stimulatory effect on BUVEC proliferation, but IP_3_-induced Ca^2+^ mobilization of intracellular stores was sufficient for BK to increase BUVEC proliferation. Notably, vasoinhibins reduced the BK-induced endothelial cell proliferation to basal levels, both in the absence and in the presence of extracellular Ca^2+^ (Figure 4) indicating first, that vasoinhibins interfere with IP_3_-induced Ca^2+^ mobilization and second, that they also affect extracellular Ca^2+^ influx to exert their inhibitory effect. We examined next the role of TRPC channels in the Ca^2+^ mobilization initiated by BK.

### 3.2. Vasoinhibins Interfere with TRPC-Mediated Ca^2+^ Entry to Inhibit BK-induced NO Production and Proliferation in BUVEC, and are Associated with Reduced TRPC5 Content

**Figure 5:**
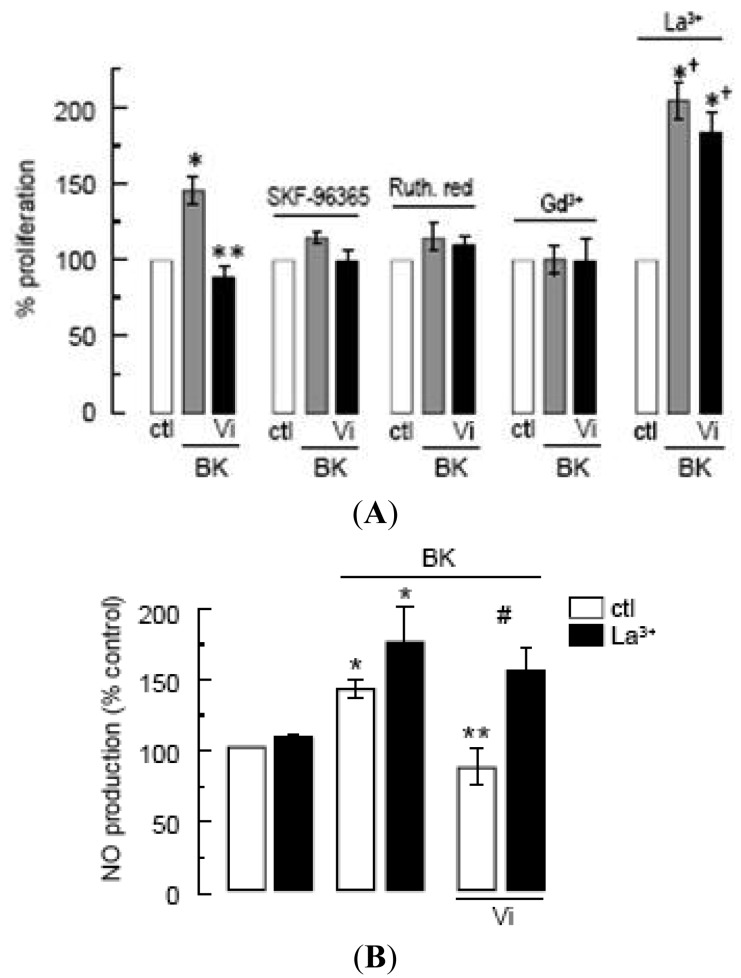
Vasoinhibins interfere with TRPC-mediated Ca^2+^ entry to modulate the BK-stimulated NO production and proliferation in BUVEC.

In the absence of specific inhibitors of TRPC channels, we tested SKF-96365, ruthenium red, and Gd^3+^, which have been extensively used to investigate the role of TRPCs [19,27,39]. These inhibitors have off-targets [39] including voltage-gated Ca^2+^ channels, which appear to be absent, however, in endothelial cells [19,38]. All three agents completely inhibited the proliferation of BUVEC in response to BK, supporting the contribution of TRPC channels to this phenomenon (Figure 5A). Under these conditions, vasoinhibins had no effect (Figure 5A). Notably, micromolar concentrations of La^3+^, known to inhibit TRPC1, 3, 6, and 7 channels [40] but to potentiate TRPC4 and 5 [20,41], enhanced the BK effect and prevented the inhibitory effect of vasoinhibins (Figure 5A). Furthermore, we observed that vasoinhibins blocked BK-induced NO production and that this effect disappeared in the presence of La^3+^ (Figure 5B). La^3+^ alone had no effect on either basal or BK-stimulated NO levels. These data suggest the involvement of TRPC4/5 in the BK-induced BUVEC proliferation. This is not unexpected since TRPC4 and TRPC5 have been shown to form nonselective cation channels, which integrate G-protein-coupled receptor signaling pathways that are activated independently of store depletion [20,42].

**Figure 6:**
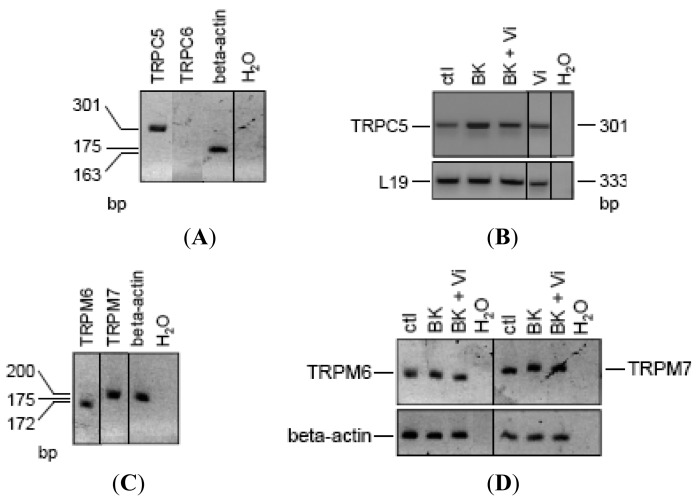
Vasoinhibins reduce the increase of TRPC5 transcript induced by BK in BUVEC.

Based on sequence, function, pharmacology, and regulatory similarities, the seven members of the mammalian TRPC family are grouped in four subfamilies; TRPC4/5, TRPC1, TRPC3/6/7, and TRPC2 [19,20]. All of them except TRPC2 have been found in endothelial cells, but their expression pattern varies according to the vascular bed [19]. Notably, TRPC6 contributes to BK-induced mobilization of Ca^2+^ in capillary- and aorta-derived endothelial cells [21], and TRPC6-deleted mice are hypertensive [43]. Also, TRPC5 [44,45], but not TRPC4 [46], is a vascular target of NO. There is no report of TRP channel expression in BUVEC. RT-PCR analysis in BUVEC showed the expression of TRPC5 but not of TRPC6 (Figure 6A). Notably, the 48-h treatment of BUVEC with BK induced an apparent increase in TRPC5 expression that was reduced by the concomitant administration of vasoinhibins (Figure 6B). Interestingly, vasoinhibins alone had no effect on TRPC5 expression. Human umbilical vein endothelial cells (HUVEC) contain other types of TRP channels, known as members of the melastatin family (TRPM), specifically TRPM7 and its closest homologue TRPM6 [47]. Because silencing TRPM7 in HUVEC increases eNOS expression and NO production [48], we examined TRPM6 and TRPM7 mRNA expression in BUVEC (Figure 6C). The expression of TRPM6 and TRPM7 was detected in BUVEC but was not modified by adding BK alone or combined with vasoinhibins (Figure 6D). Together these observations suggest that TRPC5 channels may be a specific target for vasoinhibins in reducing BK vascular effects.

### 3.3. Vasoinhibins Reduce BUVEC Proliferation by Promoting the PP2A-Mediated Inactivation of eNOS

**Figure 7:**
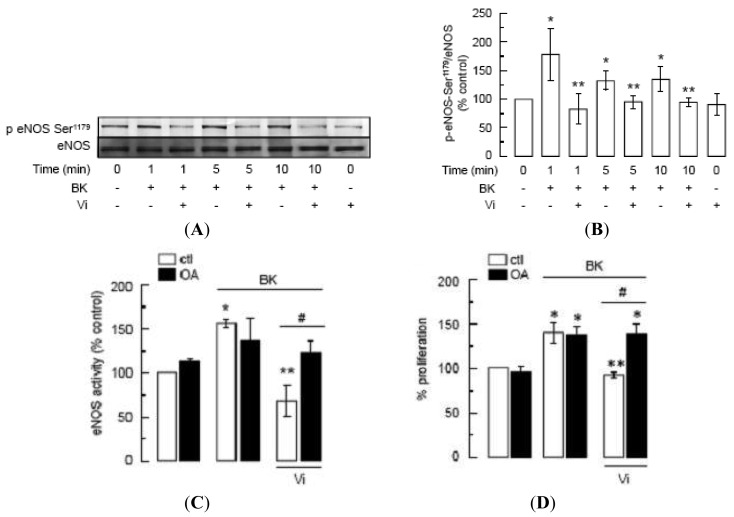
Vasoinhibins prevent the BK-induced phosphorylation of eNOS at Ser^1179^ by activating PP2A.

Phosphorylation of eNOS at Ser^1179^ renders eNOS more sensitive to Ca^2+^/calmodulin binding [23,24] and contributes to eNOS-vascular functions [49,50] and to BK signaling [22]. Western-blot analysis showed that BK enhanced eNOS phosphorylation at Ser^1179^ in BUVEC as compared to untreated cells, whereas incubation with vasoinhibins for 1 h prior to BK addition blocked BK-induced eNOS phosphorylation (Figure 7A). Phosphorylation levels in the presence of vasoinhibins alone were similar to those of untreated controls. Figure 7B shows quantification of phosphorylated eNOS after normalizing for the amount of total eNOS on the gel. Phosphorylation of eNOS occurred within 1 min after addition of BK, and the level of phosphorylation did not further increase with longer exposure to BK (5 and 10 min).

Because protein phosphatase-2A (PP2A) dephosphorylates eNOS at Ser^1179^ [15], and vasoinhibins activate PP2A in BUVEC [5], we used okadaic acid (OA), a PP2A inhibitor [51], to investigate whether the inhibition by vasoinhibins of BK-induced eNOS activity and BUVEC proliferation is mediated by PP2A. Consistent with the inhibitory effect of vasoinhibins on NO production and eNOS phosphorylation, vasoinhibins blocked BK-induced eNOS activity in BUVEC, and this effect was prevented by OA (Figure 7C). OA alone had no effect on either basal eNOS activation or BK-induced eNOS stimulation (Figure 7C). Similarly, OA abrogated the vasoinhibin effect on BK-induced BUVEC proliferation, and OA alone did not modify basal growth (Figure 7D). Altogether these data show that vasoinhibins prevent the BK-induced BUVEC growth *via* PP2A-mediated inactivation of eNOS.

## 4. Conclusions

The vascular effects of BK have been largely described and associated with disorders that include diabetic retinopathy [8-11]. The present study demonstrates that vasoinhibins, a family of endogenous peptides exerting angiostatic effects in the retina [2,3,5,52,53], block the BK-induced endothelial cell proliferation by lowering eNOS-derived NO levels *in vitro*.

The deleterious consequences of uncontrolled NO production likely contributed to the evolution of complex regulatory mechanisms to control eNOS activity. Strikingly, our current and previous work shows that vasoinhibins target several of these mechanisms to reduce NO levels. First of all, vasoinhibins block pathways participating in the increment of intracellular Ca^2+^, an event necessary for eNOS binding to calmodulin. As previously hypothesized [14], we demonstrated that vasoinhibins prevent the PLC-derived IP_3_-mediated release of intracellular Ca^2+^ stores (Figures 2 and 3) and consequently, the activation of the capacitative entry of extracellular Ca^2+^. In addition, our data identified a new target for vasoinhibins to regulate intracellular Ca^2+^ levels, the TRPC channels. Despite growing evidence that these channels participate in the regulation of angiogenesis [19], how their activity is orchestrated by the balance between pro- and antiangiogenic factors is largely unknown. Our data suggest that under long-term exposure, BK may ensure eNOS activation by increasing TRPC5 channel content, and thereby, TRPC5-mediated Ca^2+^ entry, which would enable the increase in intracellular Ca^2+^ levels necessary for eNOS activation. The eNOS-derived NO could then act as a positive feed-back messenger in the BK signaling cascade as it is able to directly activate the TRPC5 channel [45]. Furthermore, our results suggest that vasoinhibins can modulate the BK/NO/TRPC5 signaling loop by specifically reducing TRPC5 transcript content, leading to the restriction of the BK proliferative signal. It remains to be determined whether vasoinhibins could directly impede TRPC5-mediated Ca^2+^ influx, but vasoinhibin action on TRPC5 channels probably represents an additional mechanism that potently regulates BK actions on vascular homeostasis. In addition, vasoinhibins promote dephosphorylation of eNOS at Ser^1179^, a modification that reduces eNOS sensitivity to Ca^2+^-calmodulin.

This study provides new insights into the signal transduction pathway and ion channels mediating vasoinhibin angiostatic activity that could help define the nature of the vasoinhibin receptor, an issue that warrants further investigation.

## Figures and Tables

(**A**) Effect of vasoinhibins (Vi) on proliferation of BUVEC cultures stimulated or not (ctl) by BK (10 μM) for 48 h, calculated from the ^3^H thymidine incorporated as a percent of the control. Data are presented as mean ± SEM, n = 3. * *P* < 0.05 versus no treatment (ctl) and ** *P* < 0.05 versus BK. (**B**) Effect of DETA-NONOate (Det., 10 μM) on BUVEC proliferation in the presence or the absence of BK (10 μM) combined or not with Vi (60 nM). Data are presented as mean ± SEM, n = 4. * *P* < 0.05 versus no treatment (ctl); ** *P* < 0.05 versus BK; and # *P* < 0.05 versus Vi combined with BK.

PLC activity (**A**) and IP_3_ production (**B**) in BUVEC incubated for 2 min with basal medium (ctl), vasoinhibins (60 nM) or BK (10 μM), this latter being preceded or not by a 5-min incubation with vasoinhibins (Vi + BK). Data are presented as mean ± SEM, n = 3. * *P* < 0.001 versus ctl and ** *P* < 0.001 versus BK.

*Left panels* (**A**), (**C**), (**E**): In representative cells, perfusion of BK or Tg induced Ca^2+^ store depletion in a Ca^2+^-free solution, and CCE occurred immediately after addition of 2 mM Ca^2+^ to the bath solution. Intracellular Ca^2+^ concentration ([Ca^2+^]i) is represented in cells pre-incubated (black circles) or not (open circles) with either Vi or heat-denatured Vi (ΔVi) prior to BK (10 μM) or Tg (1 μM) treatment. *Right panels* (**B**), (**D**), (**F**): Histograms summarizing the magnitude of Ca^2+^ store depletion and CCE induced by BK (10 μM) or Tg (1 μM) in BUVEC in the absence (white column) or in the presence (black column) of Vi or prolactin (PRL, 10 nM). Values are normalized to untreated conditions; maximum cytoplasmic Ca^2+^ increase in BUVEC treated with BK or Tg alone is shown as reference. Data are presented as mean ± SEM, n = 3−4. * and ** *P <* 0.05 versus BK in 0 and 2 mM extracellular Ca^2+^ solution, respectively.

Cell proliferation was measured by the MTT assay after a 48-h incubation in Ca^2+^ free or in 0.4 mM Ca^2+^-containing medium, supplemented with BK (10 μM) and/or Vi (60 nM) as indicated. * *P* < 0.05 versus ctl in 0.4 mM Ca^2+^; ** *P* < 0.05 versus BK in 0.4 mM Ca^2+^; and # *P* < 0.05 versus ctl in Ca^2+^-free medium.

(**A**)Effect of SKF-96365 (10 μM), ruthenium red (Ruth. Red, 10 mM), gadolinium (Gd^3+^, 1 μM), and lanthanum (La^3+^, 10 μM) on the inhibition by Vi of BK-induced BUVEC proliferation using MTT assay. * *P* < 0.05 versus ctl; ** *P* < 0.05 versus BK; and † *P* < 0.05 versus La^3+^ alone; (**B**)Effect of La^3+^ (10 μM) on the inhibition by Vi of BK-induced NO production in BUVEC. * *P* < 0.05 versus ctl; ** *P* < 0.05 versus BK; and # *P* < 0.05 versus BK and Vi. Data are presented as mean ± SEM, n = 3.

(**A**) RT-PCR analysis of reverse-transcribed total RNA from BUVEC amplified with the TRPC5 and TRPC6 primers described in Materials and Methods. (**B**) Changes of mRNA expression in BUVEC, either untreated (ctl) or treated with BK (10 μM) in the presence or in the absence of Vi for 48 h. BUVEC analyzed for TRPM6 and TRPM7 mRNA content (**C**) and changes in expression (**D**). Normalization was based on the fact that the amounts of cDNA added to the PCR reaction are at least equal to relative normalization based on the use of housekeeping genes (beta-actin or L19). The predicted sizes of PCR products are given in base pairs (bp). Results are representative of three independent experiments.

(**A**) Western blot analysis of eNOS phosphorylation at Ser^1179^ in BUVEC pre-incubated or not with Vi (60 nM) for 1 h and then treated with BK (10 μM) for 1, 5, or 10 min. Total eNOS served as loading control. (**B**) Quantification of eNOS phosphorylation by densitometry normalized to total eNOS. Values are means ± S.E.M. of 3 independent experiments. * indicates *P* < 0.05 versus control and ** indicates *P* < 0.05 versus BK. (**C**) eNOS activity determined by the [^3^H]L-citrulline assay and (**D**) cell proliferation determined by MTT assay in BUVEC preincubated or not with okadaic acid (OA, 50 nM) for 10 min and then treated or not with BK (10 μM) in the presence or in the absence of Vi (60 nM) for 1 h. Data are presented as mean ± SEM, n = 3. * *P* < 0.05 versus no treatment (ctl); ** *P* < 0.05 versus BK; and # *P* < 0.05 versus BK and Vi.
